# *Hamacreadium cribbi* n. sp. (Digenea: Opecoelidae) from *Lethrinusminiatus* (Forster) (Perciformes: Lethrinidae) from New Caledonian waters

**DOI:** 10.1007/s11230-016-9662-8

**Published:** 2016-09-14

**Authors:** Rodney A. Bray, Jean-Lou Justine

**Affiliations:** 1 Department of Life Sciences, Natural History Museum, Cromwell Road, London, SW7 5BD UK; 2 Institut de Systématique, Évolution, Biodiversité, ISYEB, UMR7205 CNRS, EPHE, MNHN, UPMC, Muséum National d’Histoire Naturelle, Sorbonne Universités, CP 51, 57 rue Cuvier, 75231 Paris Cedex 05, France

## Abstract

A new species of *Hamacreadium* Linton, 1910, *H. cribbi* n. sp. is described from *Lethrinus miniatus* (Forster) from the waters off New Caledonia. It is compared with the other species of *Hamacreadium* reported from lethrinids and is characterised by the size of its eggs which tend to be larger [72–93 (84) *vs* 54–81 (56) µm long] than those of other species. Other characteristics, such as body size and shape and internal ratios, differentiate *H. cribbi* from other species; these differences are discussed in detail.

## Introduction

*Hamacreadium* Linton, 1910 is one of a group of plagioporine genera which are poorly defined morphologically and is ‘characterized by a combination of rather generalized plagioporine characters. Pre-eminent among those are the excretory vesicle extending into the forebody, deeply lobed ovary, distinctly submedian genital pore, and the vitelline follicles entering the forebody and extending beyond the testes to the posterior extremity’ (Cribb, 2005). The opecoelids are a group in need of a thorough molecular examination, and as a start we have sequenced a variety of worms, and have found that the *Hamacreadium*-like species found in *Lethrinus miniatus* (Forster) clusters with, but is distinct from worms identified as *H. mutabile* Linton, 1910 (see Justine et al., 2012b; Andres et al., 2014; Bray et al., 2016). To facilitate the discussion of this species in our phylogenetic studies we here describe and name this form.

## Materials and methods

Fish were collected along the external slope of the reef, off Nouméa, New Caledonia. Digeneans were collected live, immediately fixed in nearly boiling saline (Cribb & Bray, 2010; Justine et al., 2012a) and then transferred to 80% ethanol. Whole-mounts were stained with Mayer’s paracarmine, dehydrated in an ethanol series, cleared in beechwood creosote and mounted in Canada balsam. Measurements were made through a drawing tube on an Olympus BH-2 microscope, using a Digicad Plus digitising tablet and Carl Zeiss KS100 software adapted by Imaging Associates, and are quoted in micrometres. The following abbreviations are used: BMNH, British Museum (Natural History) Collection at the Natural History Museum, London, UK; MNHN JNC, Muséum National d’Histoire Naturelle, Paris, France.

**Family Opecoelidae Ozaki, 1925**

**Subfamily Plagioporinae Manter, 1947**

**Genus*Hamacreadium*Linton, 1910**^1^

***Hamacreadium cribbi*n. sp.**^2^

Syns *Neolebouria* sp. A. of Justine et al. (2010b) in part, *Hamacreadium* sp. of Bray et al. (2016)

*Type-host*: *Lethrinus miniatus* (Forster) (Lethrinidae), Trumpet Emperor.

*Type-locality*: Récif Kué, External slope, New Caledonia (22°34′892S, 166°29′673E; 21.vi.2007).

*Other localities*: Off Ever Prosperity, external slope, depth 60 m, New Caledonia (22°27′S, 166°21′E; 22.viii.2006, 11.ix.2006, 07.xi.2006).

*Type-material*: Holotype: MNHN JNC2204a. Paratypes: MNHN JNC1924, 1952, 2114, 2162, 2184, 2186, 2202, 2203, 2204b, 2205, 2207, 2208, 2300, 2402, 2706, 2822B, 2824A; BMNH 2016.4.1.1-16.

*Site in host*: Digestive tract.

*Prevalence*: 83% (24 of 29).

*Etymology*: This species is named for our colleague Tom Cribb of the University of Queensland, the pre-eminent digenean taxonomist of the Indo-Pacific Region.

### Description (Figs. 1–2)

**Figs. 1–2:**
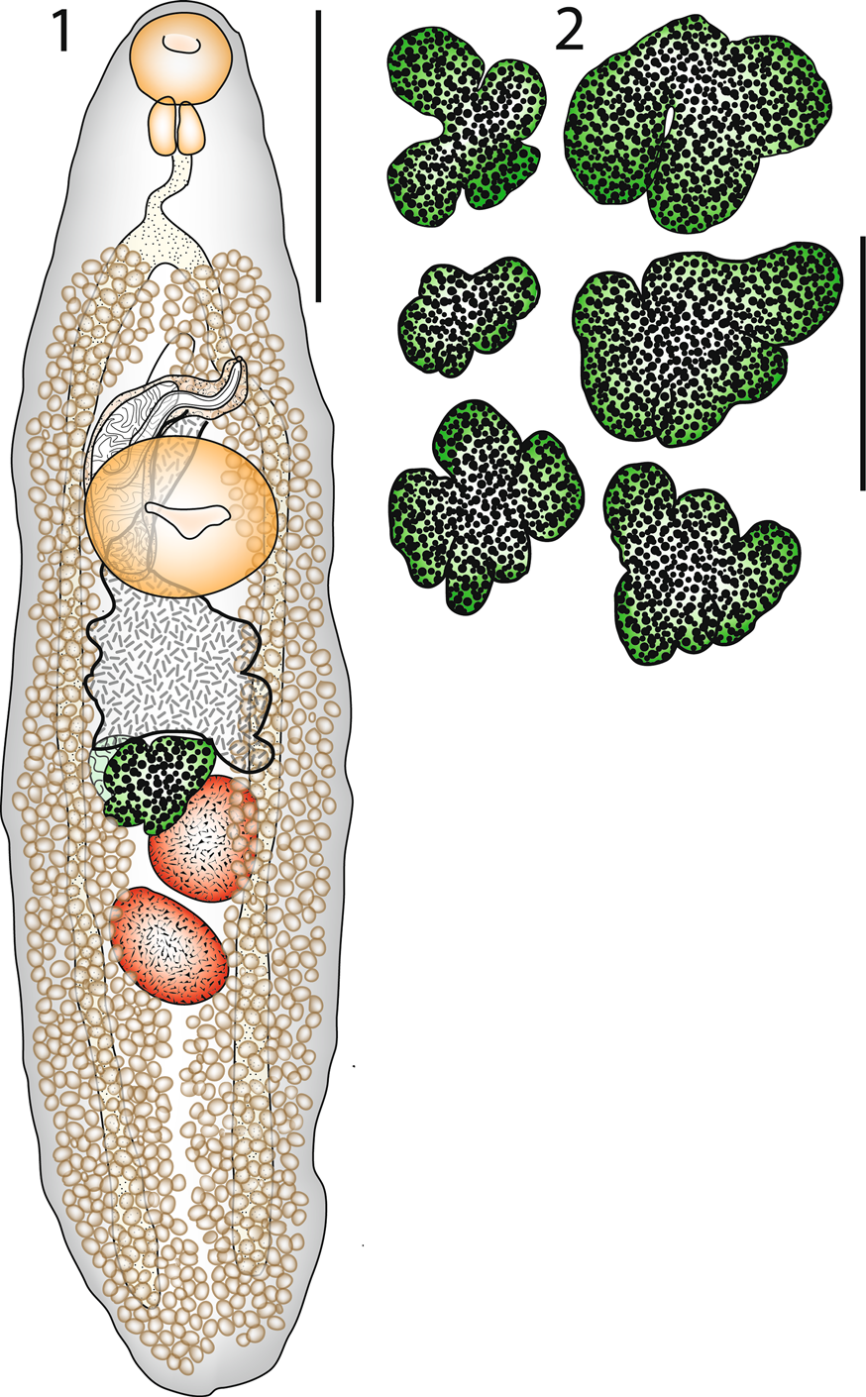
*Hamacreadium cribbi* n. sp. 1, Holotype in ventral view, with uterus outline in bold; 2, Outline drawing of six ovaries to show examples of the variation in lobation. *Scale-bars*: 1, 1,000 µm; 2, 500 µm

[Based on 90 specimens from *Lethrinus miniatus* (25 measured, see Table 1)]. Body elongate, linguiform, tapering in forebody, wider in hindbody (Fig. 1). Tegument unarmed. Pre-oral lobe distinct. Oral sucker oval, subterminal. Ventral sucker rounded, in anterior third of body, distinctly larger than oral sucker. Prepharynx short, entirely dorsal to oral sucker. Pharynx oval. Oesophagus distinct. Intestinal bifurcation in mid-forebody. Caeca narrow, end blindly in posterior quarter of post-testicular region.

**Table 1:** Measurements (in μm) and ratios of type-series of *Hamacreadium cribbi* n. sp. ex *Lethrinus miniatus* (n = 25)

Feature	Minimum	Maximum	Mean
Body length (BL)	3,049	5,747	4,585
Body width	766	1,529	1,150
Forebody length	1,138	1,935	1,517
Pre-oral lobe length	0	53	23
Oral sucker length	240	382	315
Oral sucker width	239	417	331
Prepharynx length	0	19	4
Pharynx length	140	298	212
Pharynx width	150	286	207
Oesophagus length	184	441	277
Intestinal bifurcation to ventral sucker	530	1,128	826
Vitellarium to ventral sucker	478	1,099	736
Ventral sucker length	427	690	586
Ventral sucker width	450	722	608
Cirrus-sac length	597	1,198	954
Cirrus-sac width	125	235	192
Ventral sucker to ovary	98	468	311
Ovary length	200	424	327
Ovary width	237	541	386
Ovary to anterior testis	0	2	0
Anterior testis length	301	622	445
Anterior testis width	263	573	425
Distance between testes	0	10	1
Posterior testis length	281	687	485
Posterior testis width	286	630	456
Post-testicular region	592	1,600	1,196
Post-caecal distance	84	373	201
Egg length	72	93	84
Egg width	38	56	48
Body width as % of BL	20.9	28.8	25.1
Forebody as % of BL	30.4	37.5	33.3
Sucker length ratio	1:1.53	1:2.27	1:1.87
Sucker width ratio	1:1.69	1:2.18	1:1.84
Oral sucker: pharynx ratio	1:1.23	1:1.82	1:1.61
Ventral sucker to ovary as % of BL	3.23	9.57	6.68
Post-testicular region as % of BL	19.4	31.6	25.8
Oesophagus as % of BL	4.19	9.17	6.10
Intestinal bifurcation to ventral sucker as % of BL	15.1	23.0	18.0
Vitellarium to ventral sucker as % of BL	11.5	20.4	16.1
Ovary to anterior testis as % of BL	0	0.04	0
Distance between testes as % of BL	0	0.21	0.02

Testes 2, oval to rounded, smoothly irregular, contiguous or slightly separated, in mid-hindbody, oblique. Cirrus-sac large, claviform, sigmoid, much wider proximally, reaches dorsally to ventral sucker, occasionally very slightly into hindbody, may be restricted to forebody in flattened specimens. Seminal vesicle long, coiled in posterior part of cirrus-sac. Pars prostatica and ejaculatory duct not clearly differentiated, long, coiled, complex coiling distally. Genital atrium distinct. Genital pore sinistral, ventral to left caeca or close, about halfway between bifurcation and ventral sucker.

Ovary usually lobate (3–5 lobes; see Fig. 2), oblique to and overlapping anterior testis. Seminal receptacle oval, dorsal to ovary. Mehlis’ gland pre-ovarian. Laurer’s canal opens dorsally to Mehlis’ gland. Uterus pre-ovarian, mainly intercaecal. Eggs tanned, operculate. Metraterm thick-walled, reaches level of ventral sucker. Vitellarium follicular, fields reach from bifurcal level of ventral sucker, to close to posterior extremity, lateral to caeca and encroaching slightly over dorsal and ventral surface of caeca, almost confluent in bifurcal and post-testicular regions, usually continuous at level of ventral sucker, occasionally interrupted on one or both sides.

Excretory pore terminal. Vesicle I-shaped, reaches to about level of genital pore.

### Variation

The variation seen in our specimens covers some of the features previously used to distinguish species of *Hamacreadium*:

- Vitellarium anterior extent: At level of intestinal bifurcation: 52 specimens (58%); distinctly post-bifurcal, but not to ventral sucker: 38 specimens (42%).
- Vitellarium interruptions at ventral sucker level: Continuous, no interruption: 73 specimens (81%); interruptions both sides: 7 specimens (8%); interruptions one side: 9 specimens (10%).
- Cirrus-sac reach (continuum): To about mid ventral sucker level: 49 specimens (54%); to posterior part of ventral sucker, occasionally very slightly into hindbody: 41 specimens (46%).
- Ovary lobation. Not always clear, sub-lobes complicate the picture: Three lobes: 69 specimens (77%); four lobes: 15 specimens (17%); Five lobes: 6 specimens (7%).

## Discussion

**Fig. 3:**
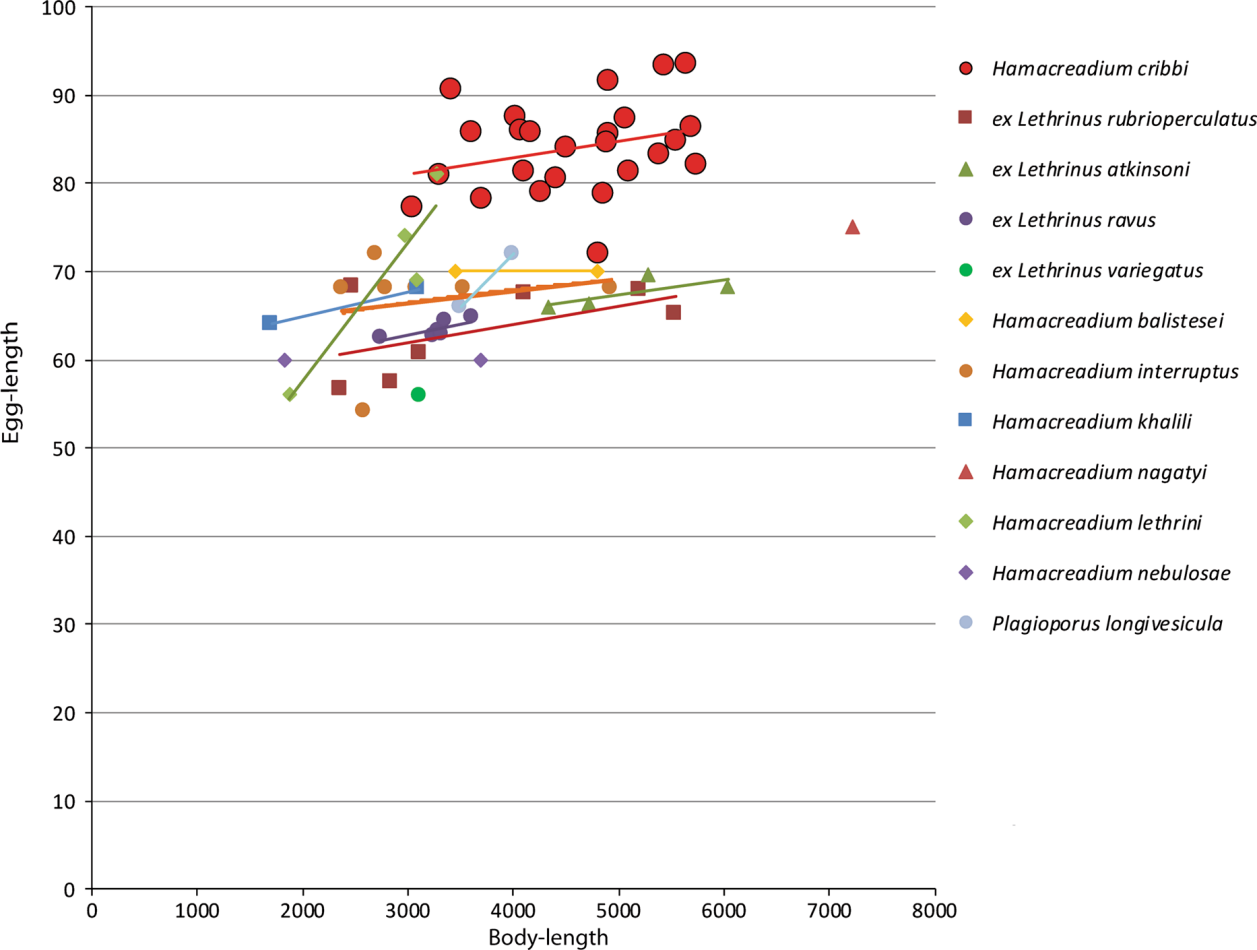
Graph showing egg-length plotted against body-length of *Hamacreadium cribbi* n. sp., original measurements of other *Hamacreadium*-like worms from New Caledonian lethrinids and measurements of named species taken from the ranges given in the literature. Values on the x and y axes are in μm

**Fig. 4:**
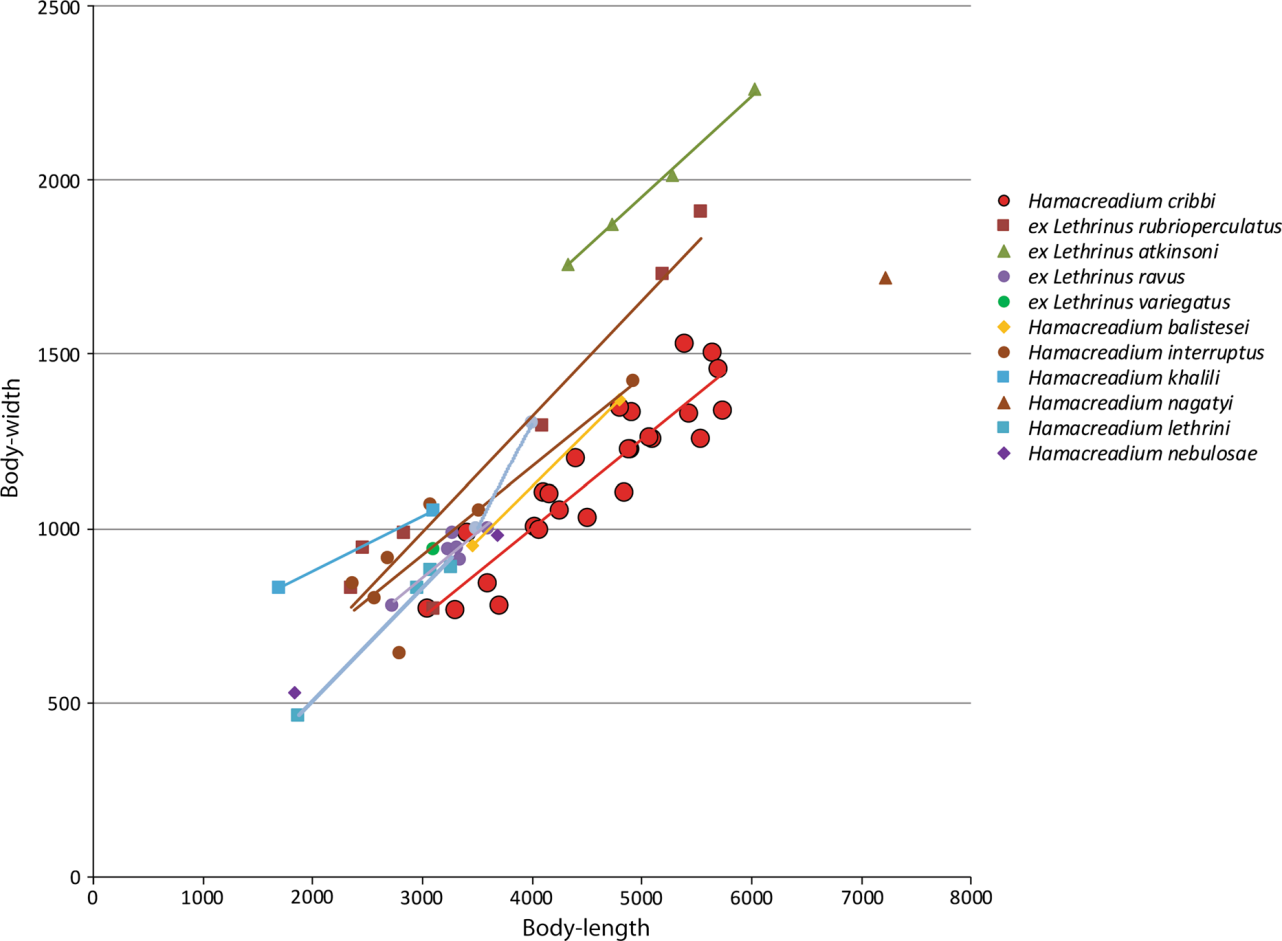
Graph showing body-width plotted against body-length of *Hamacreadium cribbi* n. sp., original measurements of other *Hamacreadium*-like worms from New Caledonian lethrinids and measurements of named species taken from the ranges given in the literature. Values on the x and y axes are in μm

**Fig. 5:**
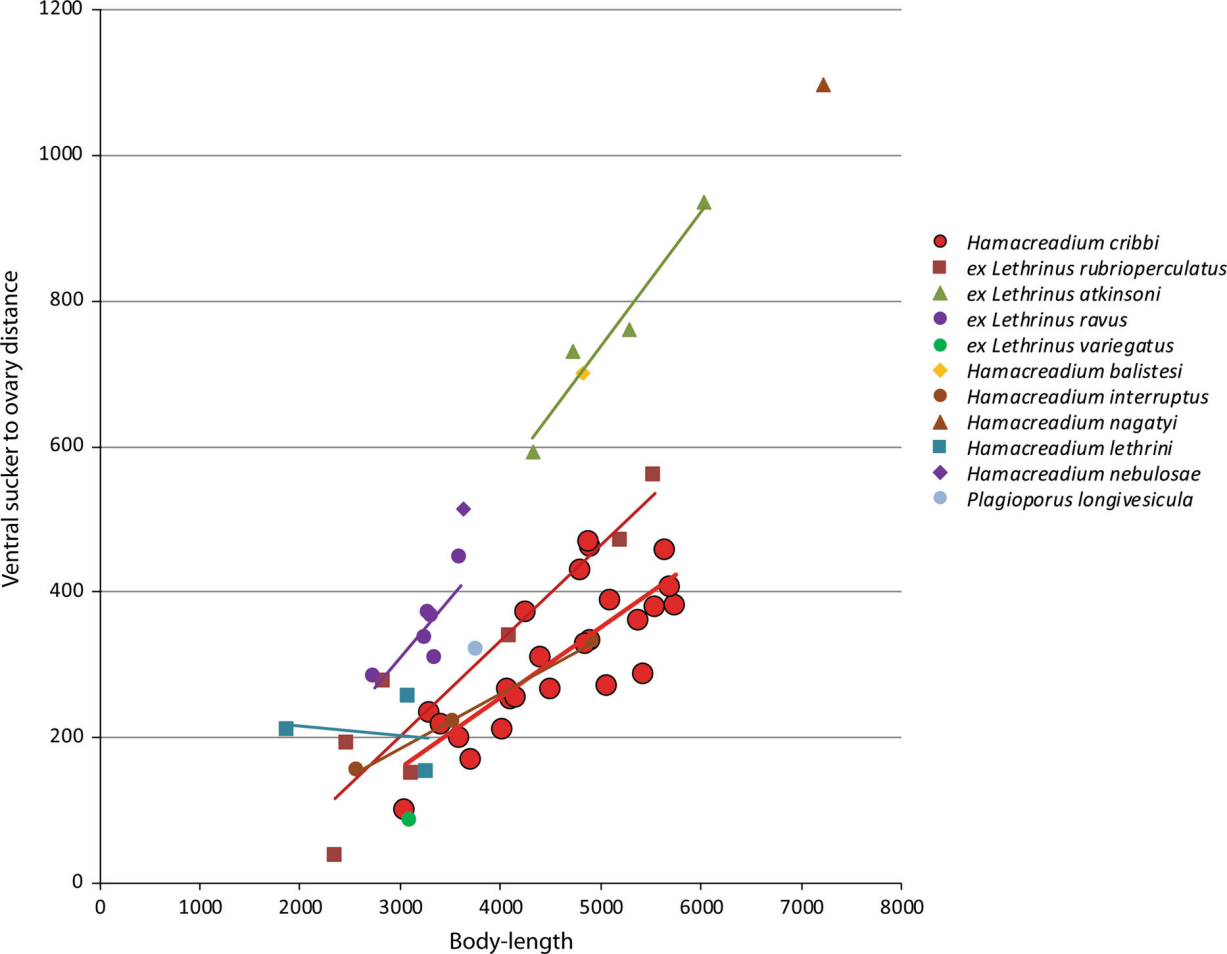
Graph showing ventral sucker to ovary distance plotted against body-length of *Hamacreadium cribbi* n. sp., original measurements of other *Hamacreadium*-like worms from New Caledonian lethrinids and measurements of named species taken from illustrations in the literature. Values on the x and y axes are in μm

This species was reported from this host species by (Justine et al., 2010b) under the name *Neolebouria* sp. A. These authors also reported the following species as hosts: the Spotcheek Emperor *Lethrinusrubrioperculatus* Sato; the Pacific Yellowtail Emperor *Lethrinus atkinsoni* Seale, the Drab Emperor *Lethrinus ravus* Carpenter & Randall, and the Slender Emperor, *Lethrinusvariegatus* Valenciennes. This description is based solely on the worms from *L. miniatus*, and it is to be noticed that worms from the other hosts may differ, possibly at the specific level. The eggs are consistently larger in the specimens from *L. miniatus* than in any of the other hosts, or indeed any of the other similar species discussed below (Fig. 3). The worms from *Lethrinus atkinsoni* are consistently broader (Fig. 4), and the relative distance between the ventral sucker and the ovary is greater (Fig. 5).

Several species of *Hamacreadium* have been reported from *Lethrinus* spp., including two reports from *L. miniatus*. Unfortunately, neither is accompanied by descriptions. Durio & Manter (1968) reported the type-species of *Hamacreadium*, *H. mutabile*, from *L. miniatus* from New Caledonia, but molecular evidence and other evidence (see below) suggests that this species occurs only in lutjanid fishes. Shen (1985) reported *H. lethrini* Yamaguti, 1934 from *L. miniatus* from off the Xisha Islands (or Paracel Islands) in the South China Sea. These species will be discussed below, along with the other *Hamacreadium* spp. from *Lethrinus* spp.

**1.*Hamacreadium balistesi*Nagaty & Abdel Aal, 1962** was originally reported from the Spangled Emperor *Lethrinus nebulosus* (Forsskål) and the White-banded Triggerfish *Rhinecanthus aculeatus* (Linnaeus) (Balistidae) from the Red Sea (Nagaty & Abdel Aal, 1962b) and was considered as synonym of *H. mutabile* by Bray & Cribb (1989). As is discussed below, *H. mutabile* is probably a specific parasite of lutjanids. The cirrus-sac in *H. balistesi* is said to be ‘preacetabular’. Nagaty & Abdel Aal (1962b) mention that the vitelline fields ‘may be interrupted at acetabular level’ and differentiate it from *H. interruptus* by, *inter alia*, ‘vitelline follicles constantly arranged or may be interrupted at acetabular level instead of their constant interrupted arrangement’, but see the Fischthal & Kuntz (1965) description of *H. interruptus* Nagaty, 1941 (discussed below). Ramadan (1983) considered *H. balistesi* a synonym of *H. interruptus*. The ventral sucker to ovary distance is relatively distinctly greater than in *H. cribbi* n. sp. (Fig. 5) and the eggs are smaller (Fig. 3). The excretory system is neither described nor illustrated.

**2.*Hamacreadium diacopae*Nagaty & Abdel Aal, 1962** was originally reported from the Dory Snapper *Lutjanus fulviflamma* (Forsskål) (Lutjanidae) from the Red Sea (Nagaty & Abdel Aal, 1962a) and later from the ‘bec de cane’ *Lethrinus* sp. (most probably *L. nebulosus*) from New Caledonia (Durio & Manter, 1968). The only description was based on a single specimen. It is small, broad (width 42% of body length, BL), with a long forebody (49% BL), the ventral sucker tends to be relatively larger [sucker-width ratio 1:1.83–2.00 (1:2.22)], it has a short post-testicular region (12% BL), the ovary overlaps the ventral sucker and the vitellarium reaches to the oesophagus. The excretory system is neither described nor illustrated.

**3.*Hamacreadiumegyptia*el Abdou, Heckmann, Beltagy & Ashour, 2001** was described from *Lethrinus nebulosus* and the Sky Emperor *Lethrinus mahsena* (Forsskål) in the Red Sea (el Abdou et al., 2001). Although reported from two hosts, measurements of only one specimen are given and these measurements vary between the description and the table. The illustrations and the tiny gonads clearly show that the worm is immature and we consider it unrecognisable.

**4.*Hamacreadium epinepheli*Yamaguti, 1934**, now known as *Cainocreadium epinepheli*, is a widely reported parasite of Indo-Pacific serranids, including those from New Caledonian waters (Bray & Justine, 2007; Justine et al., 2010a) and has been reported in the Chinese Emperor *Lethrinus haematopterus* Temminck & Schlegel, off Japan by Yamaguti (1934). Nagaty (1941) considered *H. epinepheli* a synonym of *H. mutabile*, which he reported from serranids, lethrinids and lutjanids, but later (Nagaty, 1956), recognised the species as distinct. Shen (1985) recorded *Cainocreadium epinepheli* in *L. haematopterus*, from off the Xisha Islands. The median genital pore distinguishes *Cainocreadium* from *Hamacreadium.*

**5.*Hamacreadium interruptus*Nagaty, 1941** was originally reported from the Pink Ear Emperor *Lethrinus lentjan* (Lacépède) (as *Lethrinus mahsenoides* Valenciennes) from the Red Sea (Nagaty, 1941). Fischthal & Kuntz (1965) recorded *H. interruptus* from the Smalltooth Emperor *Lethrinus microdon* Valenciennes and the Asian Swamp Eel *Monopterus albus* (Zuiew) (Synbranchidae) off Jesselton, North Borneo. They considered *Plagioporus longivesicula* Yamaguti, 1952 and *Hamacreadium lethrini* Nagaty & Abdel Aal, 1962 as synonyms. Hafeezullah & Dutta (1980) described *H. interruptus* from an unidentified marine fish off Chiria Tapu, Andaman Islands. The lack of an identified host reduces the value of the description and it is not used in comparison here. Tadros et al. (1978) reported this species from the type-host also in the Red Sea. Nagaty (1941) described and illustrated a large lateral gap in the vitelline fields at the level of the ventral sucker. Fischthal & Kuntz (1965) stated that the vitelline fields are ‘interrupted at acetabular level on both sides in 12, on left side only in 4, on right side only in 1, and uninterrupted on both sides in 3’. The eggs are smaller than those in *H. cribbi* n. sp. (Fig. 3). The excretory system is neither described nor illustrated.

**6.*Hamacreadium khalili*Ramadan, 1983** was originally reported from *L. mahsena* and *L. nebulosus* from the Red Sea (Ramadan, 1983). El-Labadi et al. (2006) reported the species from the Lavender Jobfish *Pristipomoides sieboldii* (Bleeker) (Lutjanidae) and the Yellow-edged Lyretail *Variola louti* (Forsskål) (Serranidae), from the Gulf of Aqaba in the Red Sea, without descriptive matter. The species is differentiated from *H. interruptus* in a key by ‘Testes globular and lobulated, cirrus pouch triangular’. The vitelline follicles are ‘aggregated in two sets, a posterior and an anterior, with a gap between them’. The eggs are smaller than those in *H. cribbi* n. sp. (Fig. 3). The anterior extent of the excretory vesicle was not traced.

**7.*Hamacreadium koshari*Nagaty & Abdel Aal, 1962**, originally reported in *Serranus* sp. (Serranidae) and *Lethrinus mahsena* from the Red Sea (Nagaty & Abdel Aal, 1962b), has vitelline fields restricted to the hindbody and Bray & Cribb (1989) considered it likely that it belongs on the genus *Apopodocotyle* Pritchard, 1966, now considered a synonym of *Cainocreadium* Nicoll, 1947 (see Cribb, 2005).

**8.*Hamacreadium krusadaiensis*Gupta, 1956** was originally reported from a ‘marine cat-fish’ from the Gulf of Mannar (Gupta, 1956). Hafeezullah (1971) redescribed the species from *Lethrinus nebulosus* (syn. *L. frenatus* Valenciennes) from the Gulf of Mannar off Tuticorin. Gibson (1976) transferred the species to *Neolebouria* Gibson, 1976 and it has been included in the two most recent keys to that genus (Bray & Justine, 2009; Dronen et al., 2014).

**9.*Hamacreadium nagatyi*Lamothe Argumedo, 1962** (syns *H. lethrini* Nagaty & Abdel Aal, 1962 *nec* Yamaguti, 1934; *H. lenthrium* Manter, 1963) is based on a single large specimen from *Lethrinus lentjan* (as *Lethrinus mahsenoides*) from the Red Sea (Nagaty & Abdel Aal, 1962a). As the name is pre-occupied, it has been replaced twice, by Lamothe-Argumedo (1962) and Manter (1963). At 7,220 µm long, this species is larger than any other reported lethrinid *Hamacreadium*. Cirrus-sac is entirely within the forebody. The ventral sucker to ovary distance is relatively distinctly greater than in *H. cribbi* n. sp. (Fig. 5) and the eggs tend to be smaller (Fig. 3). The excretory system is neither described nor illustrated.

**10.*Hamacreadium lethrini*Yamaguti, 1934**, was originally reported from *Lethrinus haematopterus*, from the Pacific coast of Wakayama Prefecture, Japan (Yamaguti, 1934). Fischthal & Kuntz (1964) redescribed, but did not illustrate, the species from the Longfin Emperor *Lethrinus erythropterus* Valenciennes (as *Lethrinus hypselopterus* Bleeker) and the Humpback Red Snapper *Lutjanus gibbus* (Forsskål) (Lutjanidae) from Puerto Princesa, Palawan Island. Philippines. The species has been reported two further times, but with no descriptive matter. Dyer et al. (1988) reported as host the Thumbprint Emperor *Lethrinus harak* (Forsskål) from Okinawa, Japan and Shen (1985) reported *Lethrinus miniatus* and the Grey Large-eye Bream *Gymnocranius griseus* (Temminck & Schlegel) (Lethrinidae) as hosts off Xisha Islands (or Paracel Islands) in the South China Sea. The original description is very similar to *H. cribbi* n. sp., but the testes are described and illustrated as irregularly indented, not the usual condition in *H. cribbi*. Fischthal & Kuntz (1964) described the post-testicular distance as 0–585 µm, indicating that at least one of their specimens was damaged. The eggs are generally smaller than in *H. cribbi* (Fig. 3). The excretory system reaches to about the same level as in *H. cribbi*.

**11.*Hamacreadium mehsena*Nagaty, 1941**, reported from *Lethrinus* ‘*mehsena*’ (presumably *Lethrinus mahsena*) from the Red Sea (Nagaty, 1941) differs from *Hamacreadium* in that the vitellarium does not reach into the forebody. Pritchard (1966) placed the species in her new genus *Apopodocotyle* Pritchard, 1966, but Cribb (2005) replaced in its original genus. It appears closest to the genus *Podocotyle* Dujardin, 1845, but while the testes are described as oblique, they appear almost symmetrical in the illustration. This form cannot be confused with *H. cribbi*. The excretory system is neither described nor illustrated.

**Fig. 6:**
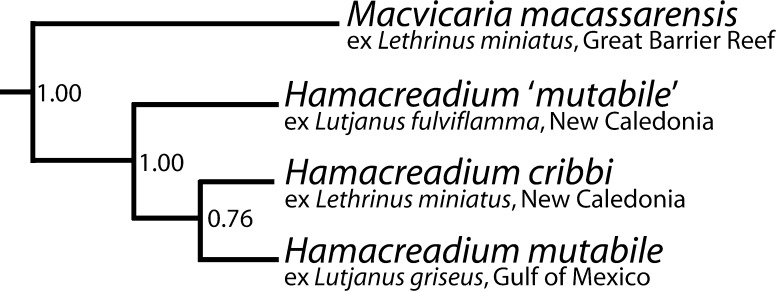
Extract from a Bayesian inference LSU + SSU rDNA tree showing the relationships of the putative *Hamacreadium* operational taxonomic units (OTUs)

**12.*Hamacreadium mutabile*Linton, 1910**, was originally described from the Grey Snapper *Lutjanus griseus* (Linnaeus) (considered the type-host), the Schoolmaster Snapper *Lutjanus apodus* (Walbaum), the Yellowtail Snapper *Ocyurus chrysurus* (Bloch) (all Lutjanidae), the Porkfish *Anisotremus virginicus* (Linnaeus) (Haemulidae) and the Gray Angelfish *Pomacanthus arcuatus* (Linnaeus) (Pomacanthidae) from the Dry Tortugas, Florida, USA, in the Gulf of Mexico (Linton, 1910). Since then it has been reported from an unfeasible number of host-species and localities, including several lethrinids. Bray & Cribb (1989) presented a long list of putative new synonyms, including *Hamacreadium balistesi*, *Hamacreadium interruptum*, *Hamacreadium lenthrium*, *Hamacreadium lethrini* Yamaguti, 1934 *nec* Nagaty & Abdel Aal, 1962, *Hamacreadium lethrini* Nagaty & Abdel Aal, 1962 *nec* Yamaguti, 1934, *Hamacreadium nagatyi*, *Hamacreadium nebulosae* and *Plagioporuslongivesicula*. The advent of molecular techniques has cast doubt on the likelihood of one species having such a wide host and locality range. Miller et al. (2011) having examined the host-specificity of fish digeneans on the Great Barrier Reef, concluded ‘that no euryxenous host distribution should be accepted on the basis of morphology only’. It is worth noting that McCoy (1929, 1930) was able to experimentally infect only lutjanids with *H. mutabile* despite the ‘numerous other species tested’. Recently, Andres et al. (2014) have registered rDNA sequence data on *H. mutabile* from *L. griseus*, from the northern Gulf of Mexico, which differs distinctly from that of *H. cribbi* (Fig. 6). *Hamacreadium mutabile* has been reported in lutjanids in New Caledonian waters (Justine et al., 2012b).

**13.*Hamacreadium nebulosae*Nagaty & Abdel Aal, 1962** is known only from three specimens found in *L. nebulosus* in the Red Sea (Nagaty & Abdel Aal, 1962b). According to the measurements given the sucker-width ratio is about 1:1, whereas in the illustration this ratio is about 1:1.8. The excretory vesicle is said to reach to the mid-level of the ventral sucker. The eggs are distinctly smaller than those of *H. cribbi* (Fig. 3).

**14.*Plagioporus*(*Plagioporus*)*longivesicula*Yamaguti, 1952** is known from two gravid and three immature specimens from *Lethrinus* sp., off Makassar, southern Sulawesi (Yamaguti, 1952). Fischthal & Kuntz (1965) considered *Plagioporus longivesicula* a synonym of *Hamacreadium interruptus* and Bray & Cribb (1989) went further and listed it as a synonym of *H. mutabile.* The excretory vesicle reaches into the pre-bifurcal region. The eggs are distinctly smaller than those in *H. cribbi* (Fig. 3).

### Molecular phylogeny

The description of this species was considered desirable as it is included in a wider study of opecoelid phylogeny and systematics based on LSU and SSU rDNA sequences. A small section of the resultant tree is included here as Figure 6 and details of the techniques are as in Bray et al. (2016). *Hamacreadiummutabile* is a widely reported parasite mainly of lutjanid fishes, and our worms from a lutjanid from New Caledonia, labelled as *Hamacreadium* ‘*mutabile*’, were identified as this species by Justine et al. (2012b). Morphologically this New Caledonian form appears practically identical to the Gulf of Mexico form, but the molecular evidence indicates that it is one of a group as closely related, but distinct, worms (Fig. 6). The Gulf of Mexico *H. mutabile* material (based only on a LSU rDNA sequence) is from the type-host in the eastern Gulf of Mexico, close to the type-locality of Dry Tortugas, Florida (Linton, 1910; Andres et al., 2014). *Hamacreadiumcribbi* clusters weakly with *H. mutabile*, it is probably more realistic to consider the *Hamacreadium* species to be related by a polytomy.

## Footnotes

1 http://urn:lsid:zoobank.org:act:504F5E58-7DB1-4AF7-8460-1514DE47DF61.

2 http://urn:lsid:zoobank.org:act:CBAC6DA3-B021-4306-9D76-3950037A8CBE
